# Habits of Eating

**Published:** 1891-12

**Authors:** 


### HABITS OF EATING.

A common cause of indigestion is irregularity respecting the time of meals. The human system seems to form habits, and to be in a degree dependent upon the performance of its function in accordance with the habits formed. In respect to digestion this is especially observable. If a meal is taken at a regular hour, the stomach becomes accustomed to receiving food at that hour, and is prepared for it. If meals are taken irregularly, the stomach is taken by surprise, so to speak, and is never in that state of readiness in which it should be for the prompt and perfect performance of its work.

The habit which many professional and business men have of allowing their business to intrude upon their meal hours, quite frequently either wholly depriving them of a meal or obliging them to take it an hour or two later than the usual time, invariably undermines the best digestion, in time. Every individual ought to consider the hour for meals a sacred one, not to be intruded upon by any ordinary circumstances.

Children should be taught to be regular at their meals, and to take nothing between meals. This rule applies to infants as well as to older children. The practice of feeding the little one every time it cries is a most serious injury to its weak digestive organs. An infant's stomach, though it needs food at more frequent intervals,—two to four hours according to its age,—requires the same regularity which is essential to the maintenance of healthy digestion in older persons. The irregularity usually practiced is undoubtedly one of the greatest causes of the fearful mortality of infants from disorders of the digestive organs, as appears in our mortuary reports.

The habit of over-eating is also responsible for a large proportion of sickness.

When more food is taken into the stomach than can be appropriated for the purpose of growth, repair, and functional activity, all the organs of digestion, assimilation, and excretion are overtaxed to dispose of this superfluity. Additional labor is put upon the kidneys, lungs, and other excretory organs, to eliminate unused material which has served no end in the human economy. And this strain long continued leads to an impairment of vigor, and not infrequently to chronic disorders which puzzle the best of physicians to overcome.

It is, therefore, a waste of energy to over-eat. But how many persons are tempted to gratify the palate long after the demands of hunger have been satisfied! It is from this class that a large percentage of invalids is recruited.

Very often the effort required in taking care of so much more food than is necessary overtaxes the whole system. A smaller quantity of nourishing food, which could be readily digested and assimilated, would give an increase of flesh and a more symmetrical roundness to the whole body. An abnormal amount of flesh, above one's average weight, is an indication of ill-health, and it may be accompanied by extreme weakness and inability to work or exercise.

We should eat to live, and not live to eat. Decide what and how much you, as an individual, need, and take that and nothing more. Put your bodily desires under the direction of the spirit, which should always hold the mastery if you hope to have health or happiness.

The average American really dines three times daily, with his beef-steak breakfast, chops for lunch, and roast beef at his dinner. And he does it at his peril, for this habit of over-feeding, especially of eating so much meat, is one of the provoking causes of so many sudden illnesses and premature deaths.

Americans are a nation of brain workers, and cannot indulge safely in high living. High thinking, or the constant use of the brain in any direction, calls for a plain but nourishing diet. Brain workers, especially, ought to live sparingly.

For the aged, or even for those above fifty, luxurious living and over-eating are specially dangerous. As functional activity lessens with increasing years, the supply of food should be decreased accordingly. An over-amount, that might be borne without disturbance in earlier years, often proves fatal in old age.

The hardiest races live on the simplest fare.

